# Epidemiological and Genetic Insights of the Circulating Foot-and-Mouth Disease Virus Serotypes in Egypt

**DOI:** 10.1007/s00284-024-03944-x

**Published:** 2024-10-30

**Authors:** Rabab T. Hassanein, Hala K. Abdelmegeed, Dina A. Abdelwahed, Abeer G. Zaki, Alaa S. Saad, Momtaz A. Shahein, Ahmed F. Afify, Mohammed A. Rohaim

**Affiliations:** 1 Virology Research Department, Animal Health Research Institute, Agriculture Research Center (ARC), Dokki, Giza, 12618 Egypt; 2 Biotechnology Research Department, Animal Health Research Institute, Agriculture Research Center (ARC), Dokki, Giza, 12618 Egypt; 3 Department of Virology, Faculty of Veterinary Medicine, Cairo University, Giza, 12211 Egypt; 4 Division of Biomedical and Life Sciences, Faculty of Health and Medicine, Lancaster University, Lancaster, LA1 4YG UK

## Abstract

Foot-and-mouth disease virus (FMDV) remains a major threat to livestock in Egypt, with ongoing outbreaks involving serotypes A, O, and SAT2. This study aimed to improve the understanding of these circulating FMDV strains to improve control measures. Between 2022 and 2023, 134 cattle samples from across Egypt were analyzed, revealing a 67.9% positivity rate for Pan FMDV. Of these positive samples, 64 were identified as serotype A and 27 as serotype O. Genetic analysis indicated that serotype O strains clustered within the EA-3 topotype, suggesting endemic persistence and potential vaccine evasion, while serotype A strains were associated with the African topotype and linked to regions such as Ethiopia, Kenya, and Sudan. Notable amino acid mutations in the VP1 protein of both serotypes highlighted potential challenges to vaccine effectiveness. These findings underscore the need for enhanced surveillance, timely vaccine updates, and regional cooperation to effectively manage FMD outbreaks in Egypt and neighboring countries.

**Supplementary Information:**

The online version contains supplementary material available at 10.1007/s00284-024-03944-x.

## Introduction

Foot-and-mouth disease virus (FMDV), a member of the Aphthovirus genus in the *Picornaviridae* family, poses a threat to livestock worldwide. This is because of its highly contagious nature, rapid transmission, leading to severe economic consequences. FMDV is a genetically diverse virus with an 8.5 Kb single-stranded RNA genome, structurally basic and lacks an envelope, and its icosahedral capsid is made up of the VP1, VP2, VP3, and VP4 proteins. Of these, VP1 is the most variable, containing critical immunogenic epitopes that are essential for eliciting protective immunity against the virus [1]. FMDV is classified into seven distinct serotypes (A, C, O, Asia1, and South African Territories [SAT] 1, SAT2, and SAT3), each of which is further subdivided into topotypes based on the VP1 sequence analysis, reflecting distinct geographical distributions and evolutionary trajectories [2]. These topotypes are based on the inherent genetic variability of the FMDV [2]. The most common serotypes are A and O, which have been reported in South America, Africa, and Asia. Currently, Africa is home to SAT1 and SAT3, while Asia 1 is found exclusively on the Asian continent. Additionally, serotype C has not caused any outbreaks since it was initially identified in Kenya and Brazil in 2004 [2]. Serotype SAT2 is particularly significant due to its ongoing presence and epidemiological impact in various regions, primarily within Africa and parts of the Middle East. SAT2 is one of the three South African Territories (SAT) serotypes, along with SAT1 and SAT3, and has been responsible for numerous outbreaks across sub-Saharan Africa [3, 4]. Unlike SAT1 and SAT3, SAT2 has exhibited a broader geographical spread and has been detected outside of Africa. Notably, SAT2 has caused outbreaks in countries like Egypt, Libya, and the wider Middle East, illustrating its ability to transcend the traditional geographic boundaries of the SAT serotypes [3, 4]. The persistence of SAT2 in endemic regions, particularly within the African wildlife-livestock interface, makes control challenging, as the virus can circulate within wildlife populations, notably African buffalo, and spill over into domestic livestock [3]. The genetic diversity within the SAT2 serotype, reflected in its multiple topotypes, complicates vaccine development and the establishment of herd immunity in affected areas. Therefore, while SAT1 and SAT3 remain largely confined to southern Africa, SAT2’s epidemiological impact extends to both Africa and neighboring regions, underscoring the importance of vigilance and control efforts to mitigate its spread.

Foot-and-mouth disease (FMD) is one of the most serious diseases impacting animals worldwide affecting around 70 species with cloven hooves, such as pigs, cattle, sheep, goats, and African buffaloes [5]. Typical FMD symptoms include fever, decreased appetite, and the formation of distinctive blisters on the feet, udders, and oral cavity [6]. Even with low mortality rates, high morbidity has a significant economic impact due to reduced production and the enforcement of trade restrictions and regional quarantine measures [7]. FMD spreads via inhaling viral particles or direct contact with infected animals’ breath. Furthermore, transmission can occur through contaminated environments, where FMDV can survive for extended periods under favorable conditions [8].

Egypt has a high prevalence of FMD; cases involving serotypes O, A, and SAT2 have been reported across the country [1, 8, 9]. Egypt first reported FMD in 1950 when serotype SAT2 first emerged. After going missing for 62 years, SAT2 emerged in 2012 causing significant losses [10]. Egypt’s SAT2 strains have been linked to the 2012 Libyan strain based on the phylogenomics and were responsible for the catastrophic outbreaks in 2018. In Egypt, serotype SAT2 is classified as topotype VII, clustering within the Alx-12 and Ghb-12 lineages [11]. Moreover, serotype O has predominant since its first report in 1951, exhibiting multiple topotypes such as the Middle East-South Asian (ME-SA) and East Africa-3 (EA-3). EA-3 first reported in 2012 and become endemic [12]. Serotype A outbreaks began in 1953, escalating in 2006 resulting in high economic losses. In Egypt, serotype A topotypes from both Asia and Africa have been reported [13, 14].

The Asian topotype of serotype A, referred to as Iran-05, was prevalent until 2010 and continued its circulation until 2015 [12, 15]. Subsequently, the African topotype emerged in early 2012, leading to the introduction of the A-Africa topotype G-IV, and continued to cause outbreaks in 2016, 2018, and 2020 [14, 16]. In 2022, routine surveillance in a single Egyptian farm identified a novel strain of FMDV serotype A, belonging to the EURO-SA lineage with genetic relationship to reported sequences identified in Venezuela and Colombia [17]. In addition, a mutant strain of FMDV/A classified as African type G-IV was reported in 2022 exhibiting a 9.3% difference in the nucleotide sequences compared to previously reported strains in 2020 [18]. These sequences were linked to the African topotype of FMD serotype A, which originated from the prototype Sudan/77 with 98.48% ± 1.2% similarity [18].

Foot-and-mouth disease (FMD) is categorized as an enzootic disease in Egypt, signifying its consistent presence within the population and its recurring nature at frequent intervals. The country is still grappled with FMD outbreaks even after deployment of various commercial vaccines. An important note is that FMD strains that are currently circulating in Egypt have been introduced by the annual importation of cattle from neighboring countries (Ethiopia, Sudan, and Somalia) [19]. There is a significant risk of introducing new variants when importing animals from areas where FMD is endemic or where various FMD lineages are prevalent, spreads to Egyptian livestock, leading to epidemics [19].

Effective management and control of foot-and-mouth disease (FMD) in Egypt requires not only the implementation of vaccination strategies but also the control of animal movements and strict biosecurity measures, monitoring the imported animals, surveillance, and collaboration with neighboring countries [20]. Through comprehensive FMD monitoring systems, widespread vaccination campaigns, and strict enforcement of biosecurity regulations, Egypt has been actively working to manage and eventually eradicate the FMD. Even in areas where vaccination campaigns are regularly conducted, occasional outbreaks still occur, despite the fact that the implementation of these control measures has resulted in a significant reduction in FMD cases. Therefore, a comprehensive strategy for FMD control and eradication must include efforts to improve the vaccination efficacy, detect and manage carriers, and strict the biosecurity measures. Egypt has implemented a comprehensive program to control the FMD, which includes vaccination using locally manufactured inactivated vaccines targeting multiple FMDV serotypes. These vaccines are tailored especially to match the virus strains that are common in particular regions [21]. However, because of RNA replication errors and inter-serotype recombination, the genetic diversity of FMDV among its seven serotypes poses a challenge [13]. Therefore, the purpose of this study is to isolate, identify, and characterize new FMDV strains by molecular means in Egyptian cattle between 2022 and 2023. This study aims to isolate, identify, and molecularly characterize the latest FMDV strains circulating in the Egyptian cattle between 2022 and 2023. This will help to improve disease surveillance, inform more targeted vaccination strategies, and promote regional cooperation for efficient FMD control and prevention.

## Materials and Methods

### Samples Collection and Preparation

A total of 134 samples were collected from 10 Egyptian governorates: Monofiya (*n* = 35), Beheira (*n* = 12), Gharbia (*n* = 14), Sharqia (*n* = 7), New Valley (*n* = 20), Sohag (*n* = 9), Minya (*n* = 23), Luxor (*n* = 3), Aswan (*n* = 4), and Beni-Suef (*n* = 7) (Supplementary Table S1). These samples were mouth epithelial (*n* = 106), heart (*n* = 14), and tongue epithelia (*n* = 14), were collected between March and December 2022 (*n* = 96) and January and April 2023 (*n* = 38) from clinically diseased cattle (*n* = 89) and buffalo (*n* = 45). Mouth epithelium samples were collected from live animals, whereas heart and tongue samples were taken from dead animals (Supplementary Fig. S1). The age and vaccination status of the animals were unspecified, as these samples were submitted to the animal health research institute (AHRI) for routine testing as part of an active surveillance program. The collected samples were collected in buffered transport medium of neutral pH following the World Organisation for Animal Health (WOAH) guidelines [22].

### Molecular Detection of FMDV

Viral RNA extraction was carried out using an EasyPure® Viral RNA/DNA Kit (TRANS, China) following the manufacturer’s instructions and RNA was kept at –80 °C until used. A total of 134 collected samples underwent screening using the PowerChek® FMD Real-time PCR Kit (Cat No. R0817E; Kogene; Korea), and the positive samples further subjected to conventional RT-PCR for virus identification and sequencing. Verso one-step Ready Mix kit (Thermo, USA) was used to perform one-step RT-PCR targeting the hypervariable region (active sites) of VP1 gene using specific primers for SAT2 [14], A [23], and O [24] serotypes.

### Sequencing and Phylogenetic Analyses

PCR products (The amplicon size was 814 bp for serotype A and 402 bp for serotype O) were purified using the QIAquick PCR Purification Kit (Qiagen) while sequencing was carried out using the BigDye® Terminator v3.1 Cycle Sequencing Kit (Life Technologies) and the DyeEx 2.0 Spin Kit (Qiagen) according to the manufacturer’s instructions. The resulting sequences were analyzed using the BioEdit program version 7.1.5. Nucleic acid and amino acid sequence similarities were analyzed using the Basic Local Alignment Search Tool (BLAST) (https://blast.ncbi.nlm.nih.gov/Blast.cgi). The Sequence Demarcation Tool (SDT) was utilized to calculate nucleotide pairwise identity scores, which were visually represented in a color-coded matrix [25]. Molecular Evolutionary Genetics Analysis (MEGA) version X was used [26], and the phylogenetic trees were constructed using the Maximum Likelihood (ML) method via RaxML version 8.2.11 [27]. To determine the most appropriate nucleotide substitution model, the general time-reversible (GTR) model, accounting for gamma-distributed rate variation among sites, was selected based on results from jModelTest [28].

### Virus Isolation and Antigen Detection ELISA

Based on the results of the serotyping molecular analysis, six representative samples (*n* = 6) were selected for viral isolation, with three belonging to serotype A and three to serotype O. FMDV isolation was carried out following the guidelines outlined by the World Organization for Animal Health, utilizing the Baby Hamster Kidney cell line (BHK-21) [29]. Cells were cultured in minimum essential medium (MEM) with Earl’s salts supplemented with 10% fetal calf serum (FCS), 100 IU/ml penicillin, and 100 mg/ml streptomycin. FMDV was passaged three successive blind passages, and the infected cells were harvested upon the appearance of cytopathic effect (CPE). Solid phase ELISA was used to detect FMDV antigens in the tissue culture suspensions of the isolated samples, and virus serotyping was employed to differentiate between O, A, C, Asia1, SAT1, and SAT 2 serotypes. The ELISA assay was carried out in compliance with the guidelines provided by the manufacturer (IZSLER, Brescia, Italy).

## Results

### Detection of Pan FMDV and Serotyping of FMDV Strains

The collected samples were subjected to an initial screening for FMDV using quantitative reverse transcription-polymerase chain reaction (RT-qPCR). Our results showed that 91 out of 134 samples, approximately 67.9%, were positive for Pan FMDV (Supplementary Table S1) with cycle threshold (Ct) values ranged from 17 to 29. Using a serotype-specific RT-PCR assay targeting the capsid-coding region, FMDV serotyping was conducted. Specifically, 27 out of 91 positive FMD samples (27/91; 29.67%) were positive for FMDV serotype O, however, 64 out of 91 positive samples (64/91; 70.3%) were positive for FMDV serotype A. These results indicate the co-circulation of FMDV serotypes A and O (Supplementary Table S1).

### Sequencing and Phylogenomics

**Fig. 1:**
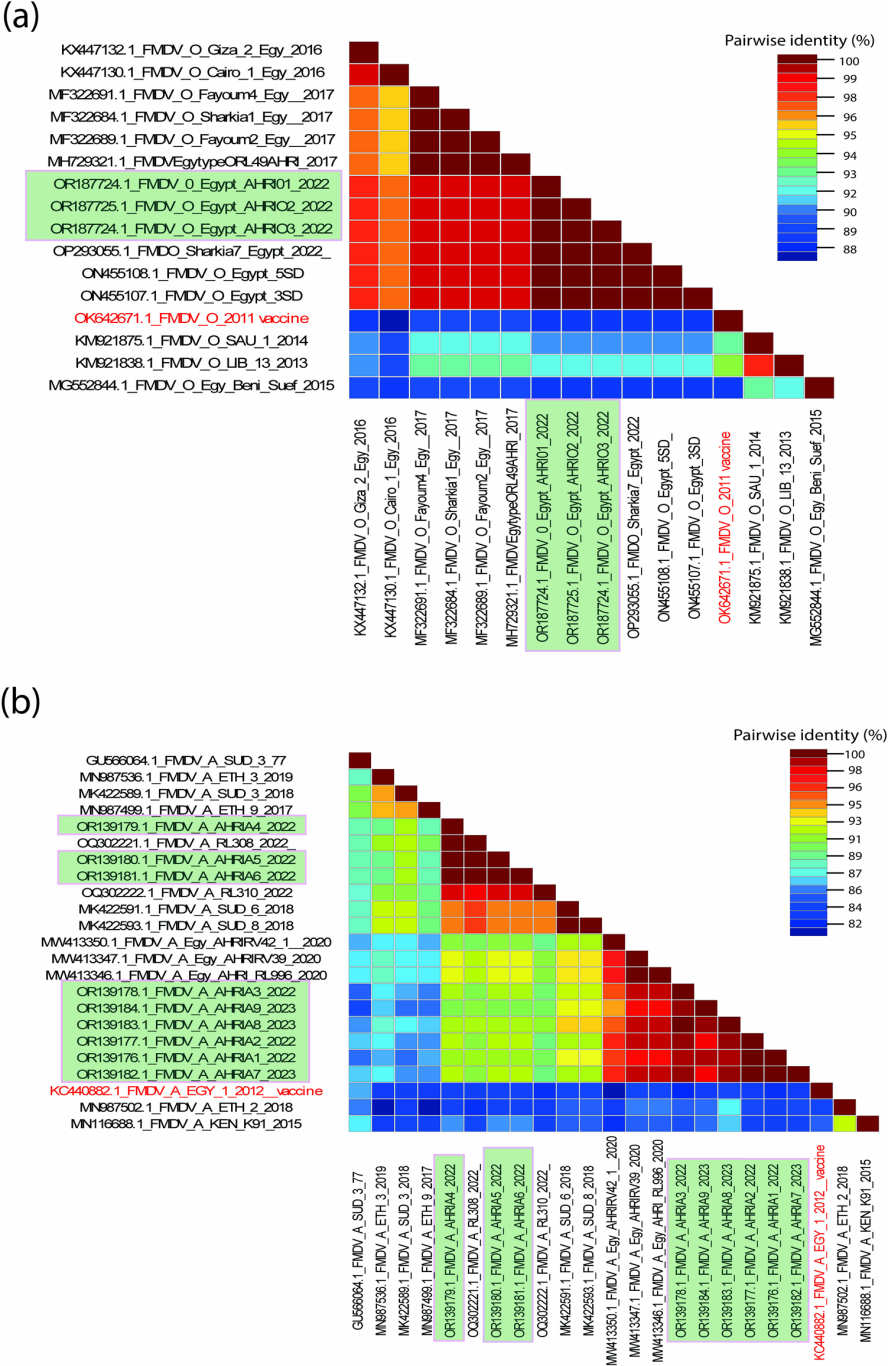
Pairwise Identities Plot of the VP1 Gene for Foot-and-Mouth Disease Virus (FMDV) Serotypes O and A. **a** The pairwise identity percentages for FMDV serotype O are shown in a plot generated by the Sequence Demarcation Tool (SDT) software. Sequences were aligned using the ClustalW algorithm, and identity values are depicted in the form of pink boxes. **b** The pairwise identity percentages for FMDV serotype A are similarly represented. The alignment and analysis methods remain consistent with those used for serotype O, with identity scores displayed in pink boxes. These plots illustrate the genetic variability within the VP1 gene across different isolates of FMDV, emphasizing the degree of sequence similarity between the viral strains for each serotype. The ClustalW alignment aids in determining regions of conservation and divergence within the sequences, which may have implications for vaccine design and viral epidemiology

**Fig. 2:**
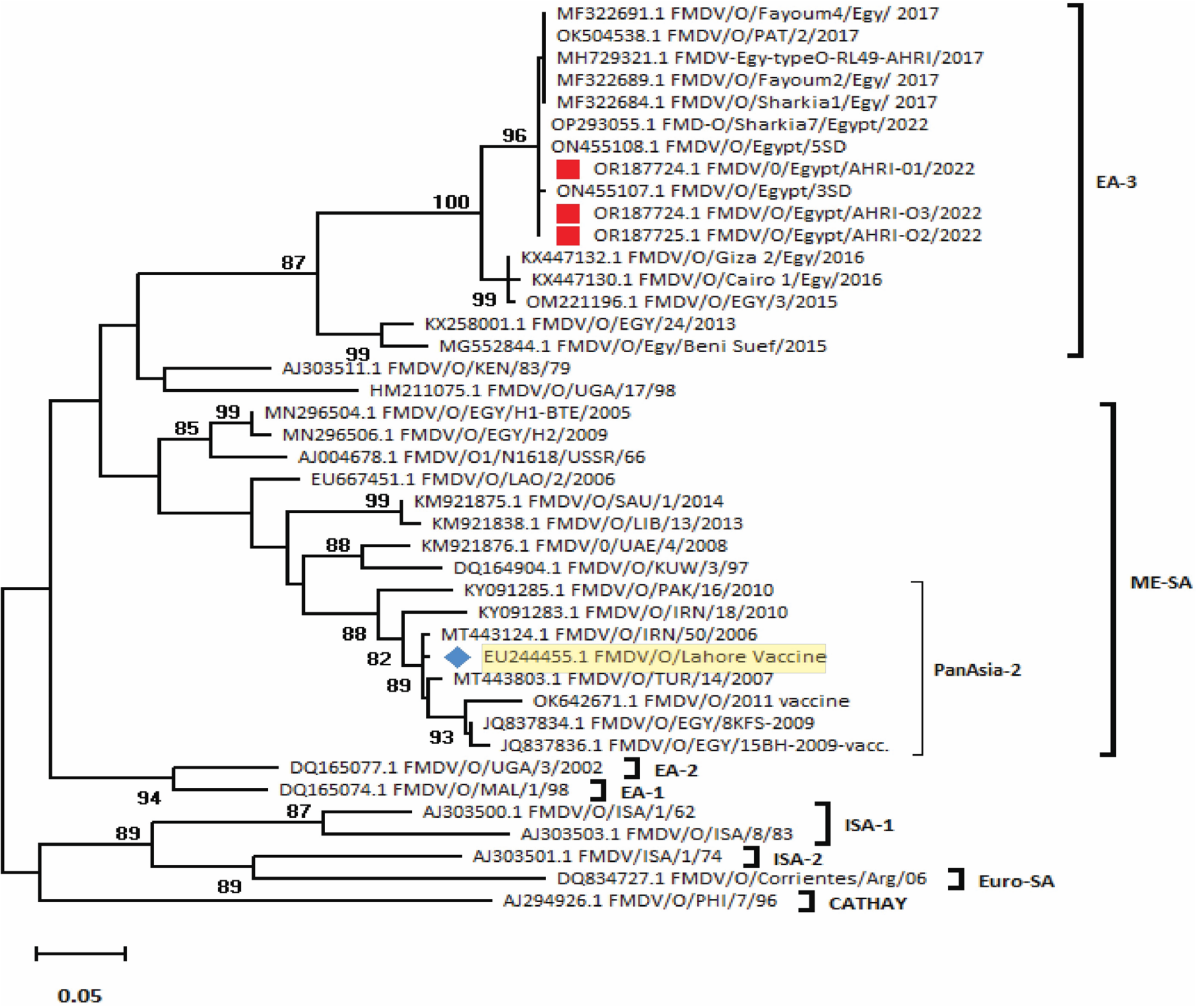
Phylogenetic Tree Based on the VP1 Gene of Foot-and-Mouth Disease Virus (FMDV) Serotype O. The phylogenetic relationships among the FMDV serotype O isolates from the current study are shown alongside reference strains. The isolates from this study are marked with red squares. Phylogenetic analysis was conducted using the VP1 gene sequences, highlighting the evolutionary relationships between the newly identified strains and known reference sequences. This tree illustrates both the genetic diversity within serotype O and the evolutionary proximity of the current isolates to other global FMDV strains. Bootstrap values (shown at the nodes) provide statistical support for the branching patterns observed, with higher values indicating greater confidence in the inferred relationships

**Fig. 3:**
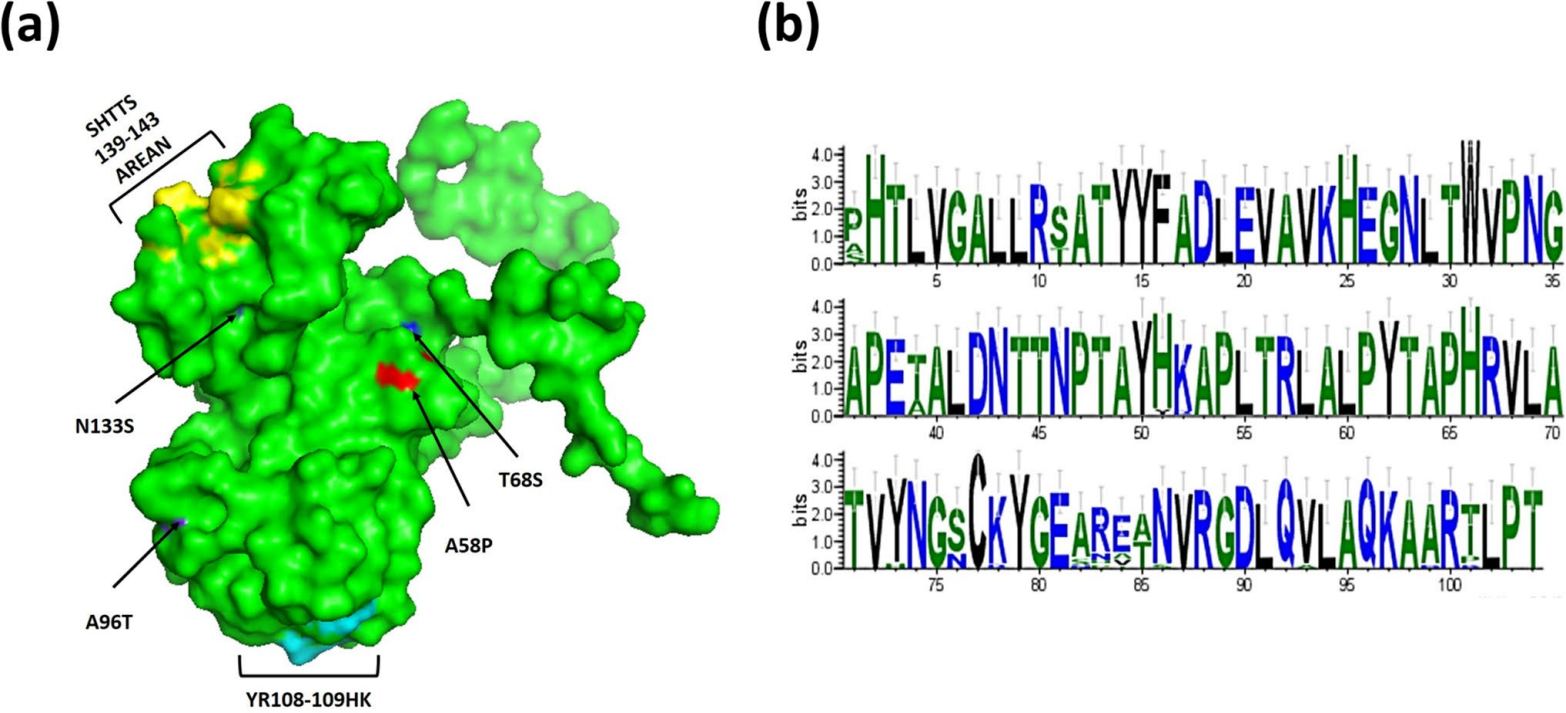
Foot-and-Mouth Disease Virus (FMDV) Mutations Trend Analysis. **a** Predicted 3D Structures of VP1 Protein in FMDV Serotype O: This panel shows a comparative visualization of the VP1 protein 3D structures for different FMDV serotype O isolates, highlighting amino acid substitutions at key residues. These substitutions are presented in comparison with the reference vaccine strain (OK642671.1 FMDV serotype O isolate FMDV/O/2011). Critical residues where mutations have occurred are marked, with differences between the isolates and vaccine strain clearly indicated. **b** Amino Acid Divergence in FMDV Serotype O Isolates: WebLogo graphs represent the sequence variation in the VP1 protein among the FMDV serotype O isolates analyzed in this study. The height of the letters at each position indicates the conservation of amino acids, with variable regions reflecting the divergence from the reference vaccine strain

The amino acid sequence identity between the studied FMDV serotype O isolates ranged from 88 to 100% (Fig. 1a) and from 82 to 100% for serotype A isolates (Fig. 1b). These isolates showed varying degrees of genetic divergence from the vaccine strains. Representative positive samples for FMDV serotype O samples (*n* = 3) were sequenced and submitted to GenBank with accession numbers OR187724–OR187726. To determine the epidemiological clustering of the Egyptian FMDV strains, representative FMDV genome sequences from the National Center for Biotechnology Information (NCBI) databases were downloaded for phylogenetic and comparative genomic analyses. A Bayesian consensus phylogenetic analysis, verified using the neighbor-joining method, revealed that three serotype O strains clustered within previously reported Egyptian isolates within topotype EA-3 (Fig. 2). Moreover, amino acid comparisons with the vaccine strain (OK642671.1 FMDV serotype O isolate FMDV/O/2011) and other reference strains revealed significant substitutions in the antigenic epitopes within the VP1 protein (Fig. 3a) that could lead to neutralizing escape variants. The webLogo graph showed multiple amino acid divergence between the FMDV serotype O isolates reported in this study and the Egyptian local vaccine strain (Fig. 3b). The identification of the tripeptide sequence _145_RGD_147_, known for its role in neutralizing determinants, underscores the significance of specific amino acid motifs in vaccine antigenicity. The G–H loop contains an RGD tripeptide which mediates host cell binding of FMDV via an integrin host cell receptor specifically, the presence of peptides like _146_GDLGSLA_150_, which coincide with trypsin-sensitive sites, underscores the importance of these regions in viral infectivity and immune recognition. Amino acid substitutions observed within the GH loop of our FMDV serotype O strain at positions 139- 143 compared to the FMDV serotype O vaccine (Supplementary Fig. S2).

**Fig. 4:**
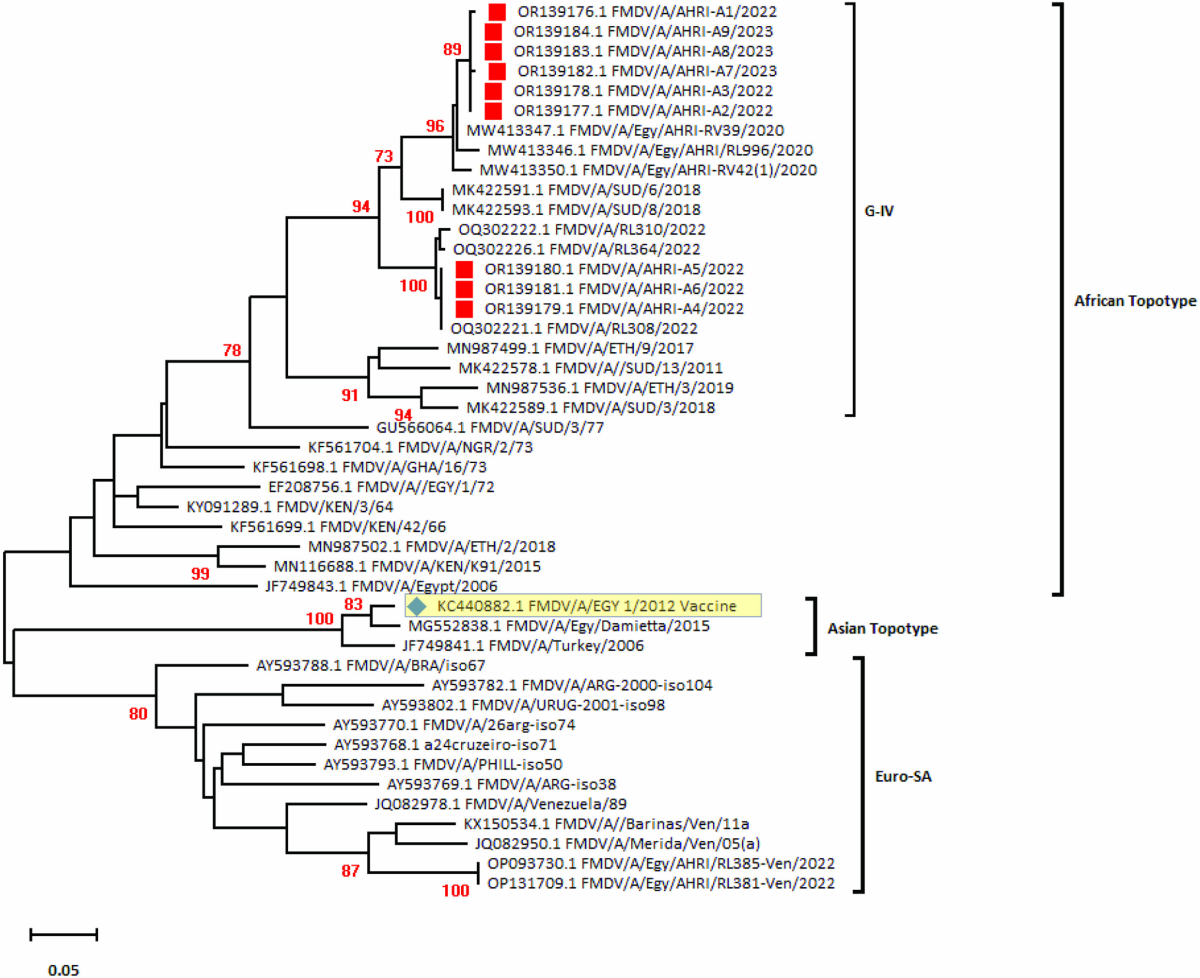
Phylogenetic relationships among three FMDV serotype A strains isolated in the current study, marked with red squares, in comparison to other global reference strains. The phylogenetic tree was constructed using the VP1 gene sequences, which is a key genetic marker for distinguishing FMDV strains. Clustering patterns suggest the evolutionary relatedness between the studied isolates and other regional strains, helping to trace lineage and mutation trends relevant to vaccine efficacy

**Fig. 5:**
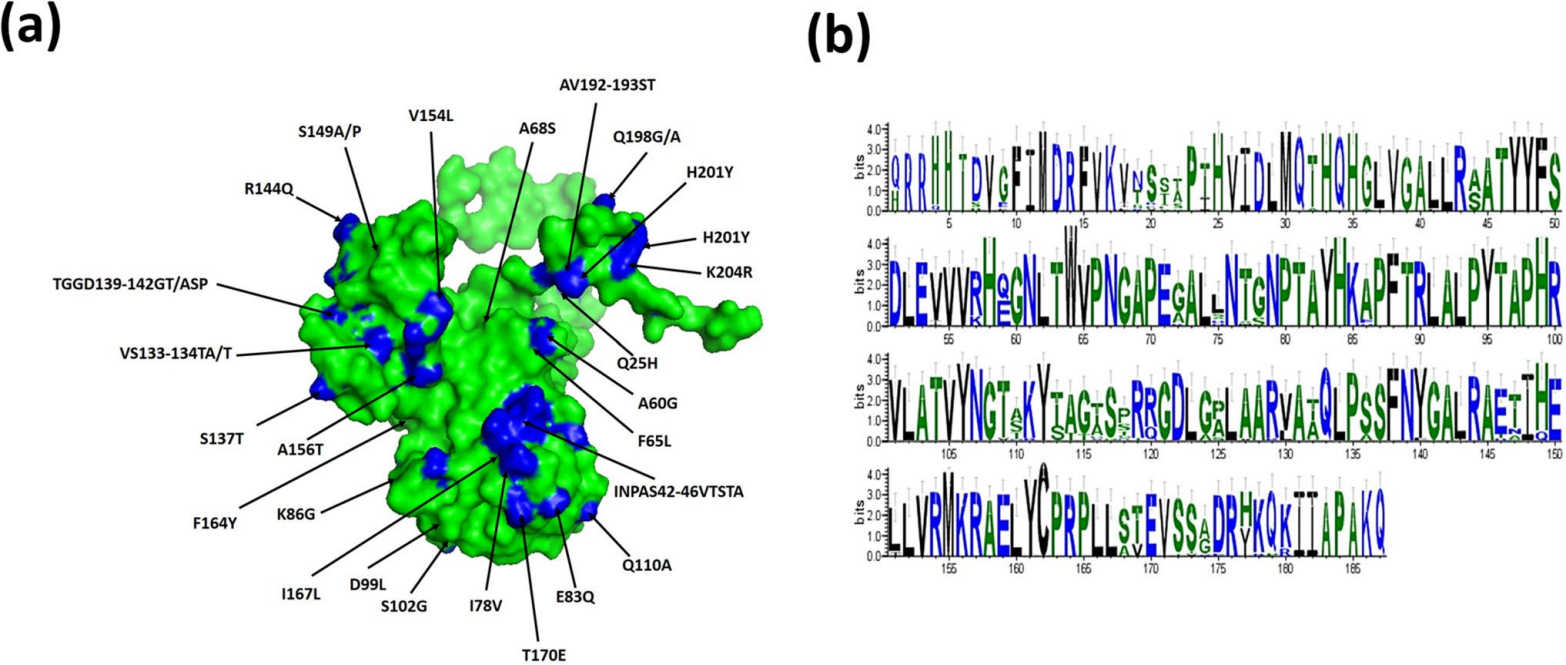
Foot-and-Mouth Disease Virus (FMDV) Mutations Trend Analysis. **a** Predicted 3D Structures of VP1 Proteins in FMDV Serotype A. The panel presents 3D structural models of the VP1 protein from different FMDV serotype A isolates. These models highlight critical amino acid residues where mutations have occurred, comparing them to the reference vaccine strain (KC440882.1 FMDV serotype A isolate EGY 1/2012). Key sites of variation between the studied isolates and the vaccine strain are indicated to demonstrate how structural changes may influence antigenicity and immune recognition. **b** The WebLogo graphs depict the sequence variability of VP1 proteins among FMDV serotype A isolates analyzed in this study. The graphs illustrate conserved and variable positions, with the height of each letter indicating the frequency of specific amino acids at each position. Divergence from the reference strain is emphasized in regions where mutations could impact the efficacy of current vaccines or immune responses

On the other hand, nine positive samples for FMDV serotype A strains have been sequenced and submitted to GenBank with accession numbers OR139176–OR139184. Phylogenetic analyses showed that these nine isolates clustered within Genotype IV of the African topotype and were closely related to the previously reported Egyptian serotype A strains in 2020, and 2022 (Fig. 4). Additionally, amino acid comparisons with the vaccine strain (KC440882.1 FMDV type A isolate EGY 1/2012) and other reference strains showed amino acid substitutions within the VP1 protein (Fig. 5a). Moreover, the webLogo graph indicated multiple amino acid divergence between the FMDV serotype A strains reported in this study compared to the Egyptian local vaccine strain (Fig. 5b and Supplementary Fig. S2). The hypervariable regions of FMDV (Foot-and-Mouth Disease Virus) serotype A are typically located within the VP1 protein, specifically in the GH loop and C-terminus regions. The GH loop, which is a major antigenic site, is found around positions 134–158 of the VP1 protein [29]. Notably, amino acid substitutions observed within the GH loop of our FMDV serotype A strain at positions 134, 135, 138, 140, 142- 154, 150, 155, and 156, as compared to the FMDV serotype A vaccine, suggest potential alterations in antigenic epitopes and binding affinity, which may influence vaccine effectiveness (Supplementary Fig. S3). Overall, providing a comprehensive analysis of amino acid mutations and their functional implications would enhance the manuscript’s contribution to understanding antigenic diversity and vaccine development strategies against FMDV.

### Virus Isolation and Antigen Detection

**Fig. 6:**
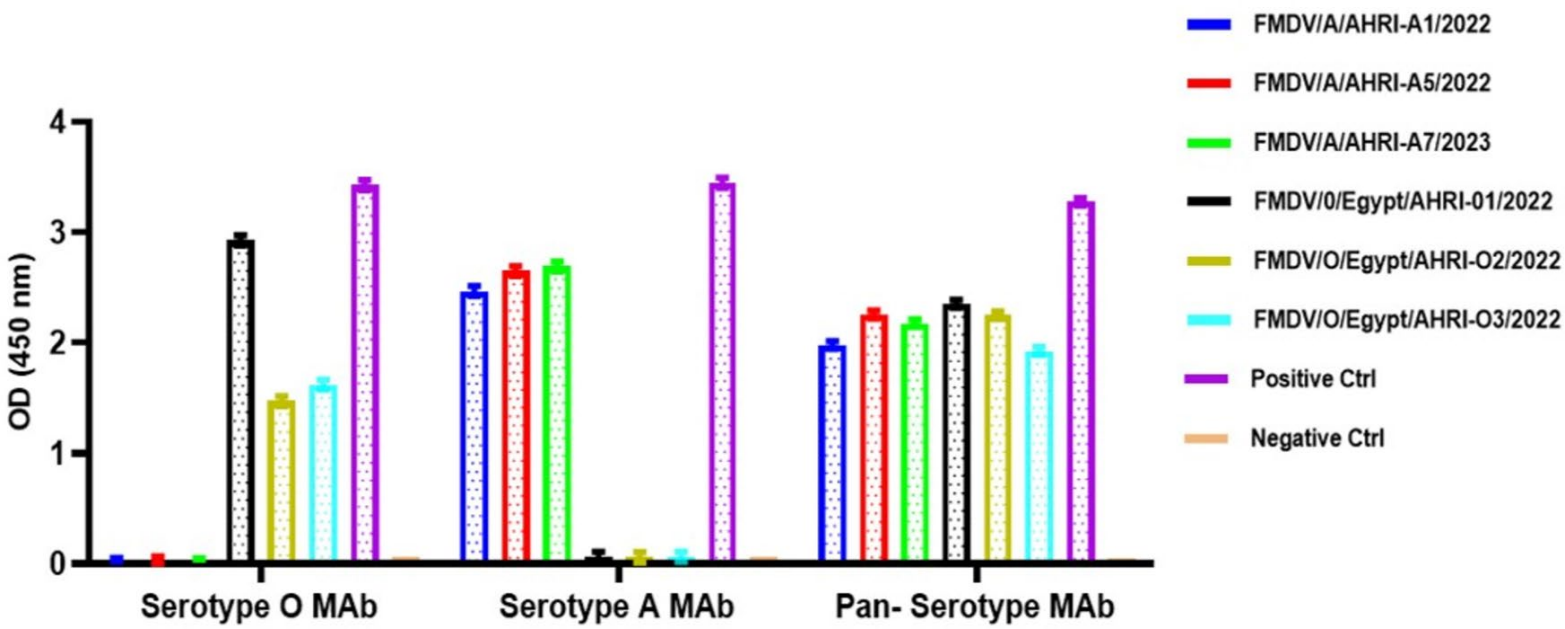
Results of the Foot-and-Mouth Disease Virus (FMDV) antigen detection ELISA. All six samples tested positive for the FMDV Pan-serotype, which included three isolates of serotype A and three isolates of serotype O. The ELISA demonstrates successful detection of FMDV antigens across both serotypes, highlighting its utility for broad-spectrum FMDV identification

Six samples were subjected for virus isolation, where the cytopathic effects (CPEs), such as rounding in cells and subsequent sloughing were observed 18–72 hours post infection (hpi) compared to non-infected cells (Supplementary Fig. S4). The third passage for all samples was subjected to antigen detection ELISA to verify the presence of the target antigen and ensure that high standards of accuracy and reliability in the isolation process. These six samples were positive toward the anti-pan-FMD antibodies. Additionally, FMDV serotype A (3 samples) showed positive reactions with serotype A-MAbs, and FMDV serotype O (3 samples) exhibited positive responses in serotype O-Mabs (Fig. 6). These results confirmed the presence of FMDV serotype A and O antigens in the tissue culture suspensions.

## Discussion

Foot-and-mouth disease virus (FMDV) is a transboundary animal disease that contributes to the emergence of diverse viral strains across different continents, posing significant challenges for control [30–33]. In Egypt, the virus is characterized by its high genetic variability, which complicates vaccine development and necessitates continuous surveillance [14]. This study aimed to provide a detailed molecular characterization of the FMDV strains currently circulating in Egypt, focusing on serotypes O and A, which have been responsible for recent outbreaks [8, 9]. Our analysis revealed that the FMDV serotype O isolates in this study exhibited 100% nucleotide identity with previously identified strains such as FMD-O/Sharkia7/Egypt/2022, FMDV/O/Egypt/5SD, and FMDV/O/Egypt/3SD. These findings suggest a recent common ancestor and indicate that the virus has been circulating with limited genetic changes. Despite this, the observed differences in the antigenic sites of the VP1 protein between these isolates and the vaccine strain raise concerns about the potential for immune escape, which could contribute to new outbreaks [18]. The serotype O isolates also showed high genetic similarity (95%) with FMDV strains from neighboring countries such as Sudan and Libya [34]. This high degree of similarity underscores the interconnectedness of FMDV transmission within the African continent and highlights the importance of regional cooperation in disease surveillance and control. The close genetic relationship between FMDV strains across these countries suggests that transboundary movements, possibly through the trade of live animals, play a significant role in the spread of the virus. These findings emphasize the need for coordinated control measures, including stringent animal movement restrictions and harmonized vaccination strategies across borders.

Similarly, our analysis of FMDV serotype A isolates revealed 97–98% nucleotide identity with Egyptian strains reported in 2020, such as FMDV/A/Egy/AHRI-RV39/2020, FMDV/A/Egy/AHRI/RL996/2020, and FMDV/A/Egy/AHRI-RV42(1)/2020. The genetic similarities indicate that these strains belong to the African topotype G-IV lineage, specifically the genotype IV that originated from the Sudan type (FMDV/A/SUD/3-77). The emergence and dominance of this genotype in 2020 reflects ongoing viral evolution and adaptation within the region. Our findings also showed lower nucleotide similarities with older strains from Ethiopia and Kenya, suggesting that while there is some genetic continuity, significant evolutionary divergence has occurred. This divergence may alter the antigenic profile, potentially reducing vaccine effectiveness, potentially diminishing the effectiveness of existing vaccines that were designed based on older strains.

Notably, the high degree of genetic similarity between FMDV strains isolated from cattle and buffalo within the same geographical areas. Specifically, the FMDV serotype O isolates exhibited an identity percentage ranging from 96 to 99%, while the serotype A isolates showed a sequence identity of 94–98%. The high similarity suggests circulation within a shared transmission network rather than forming distinct species-specific populations. This finding is significant for disease control, as it underscores the need for integrated vaccination strategies that cover all susceptible species within a region. The genetic overlap suggests that cattle and buffalo may serve as mutual reservoirs, facilitating the continued spread of the virus even in the presence of vaccination efforts.

The results regarding FMDV strains identified in 2022 and 2023 in this study have important implications for the development of vaccines and preventative measures. Given the significant degree of similarity between the isolates of FMDV serotypes O, A, and other previously reported strains, as well as their observed genetic stability, it appears that particular viral lineages are persistent in the area. Additionally, the clustering of some isolates with strains from earlier years suggests that the FMDV is still circulating and might be undergoing an evolutionary trend. Furthermore, to guarantee the vaccination’s effectiveness against circulating variations, continuous surveillance is crucial, as indicated by the conserved antigenic sites between the isolated strains and the vaccine strain. These results highlight the need for strong control measures, flexible vaccination plans, and close observation in order to handle the changing FMDV situation in Egypt. Stakeholders can successfully minimize the impact of FMD outbreaks and strive toward disease control and eradication by customizing vaccination programs and biosecurity measures based on their understanding of the genetic diversity and antigenic features of isolated strains.

One of the critical challenges identified in this study is the variation within the antigenic sites of both serotype A and O isolates compared to the vaccine strains. These variations, which include non-conservative mutations and amino acid substitutions in key regions of the VP1 protein, may affect the virus’s ability to evade the immune response induced by vaccines. This antigenic mismatch highlights the need for ongoing surveillance to monitor the evolution of circulating strains and to update vaccines accordingly. The inclusion of both vaccinated and unvaccinated samples in our study was an essential aspect, as it allowed us to assess the potential impact of vaccination on the genetic diversity of the virus. However, attributing outbreaks solely to vaccine failure requires further experimental validation, such as challenge studies, to determine the actual efficacy of the vaccines used.

Due to resource constraints, we were unable to sequence all the positive samples collected during the study. Instead, we strategically selected a subset of samples based on criteria such as geographical distribution, serotype, and viral load (indicated by Ct values). This approach allowed us to capture the broadest possible genetic diversity within the limits of our resources, although it meant that some regions and serotypes were underrepresented in the sequencing process. The samples selected for sequencing were collected from 10 different governorates across Egypt, with a focus on areas known to be FMDV hotspots or where outbreaks had been reported. This targeted selection maximized the relevance and impact of our findings, providing valuable insights into the genetic diversity of FMDV strains circulating during the study period.

In conclusion, our study provides a comprehensive molecular characterization of FMDV strains currently circulating in Egypt, with significant implications for vaccine development and disease control strategies. The high degree of genetic similarity between strains from different regions and host species underscores the interconnectedness of FMDV transmission networks and highlights the need for coordinated, region-wide control measures. The observed antigenic variations within the VP1 protein of both serotype A and O isolates point to potential challenges in maintaining vaccine efficacy, emphasizing the importance of continuous surveillance and vaccine updates. While our study provides valuable insights, it also highlights the need for further research to address gaps in our understanding of FMDV epidemiology, including the direct evaluation of vaccine efficacy through challenge studies and the exploration of other factors that may contribute to FMD outbreaks, such as host immune responses and environmental conditions.

## Supplementary Information

Below is the link to the electronic supplementary material.

Supplementary file1 (DOCX 3770 KB)

## Declarations

### Research Involving Human and Animal Participants

The study did not involve human participants and/or vertebrate animals.
