# Supplementary material for: Epidemiological and Genetic Insights of the Circulating Foot-and-Mouth Disease Virus Serotypes in Egypt
Source: Curr Microbiol. 2024 Oct 30;81(12):435. doi: 10.1007/s00284-024-03944-x (PMC11525254; doi:10.1007/s00284-024-03944-x)
Supplement: Supplementary file 1 — Supplementary file1 (DOCX 3770 KB) [file 284_2024_3944_MOESM1_ESM.docx]

**Supplementary Materials:**


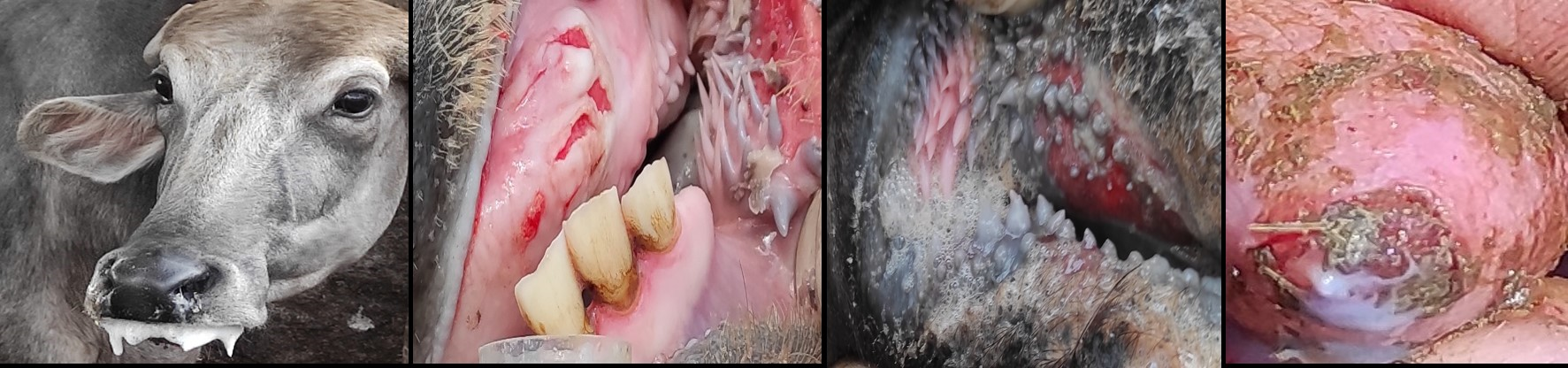


Fig. S1. Infected animals with FMDV showing salivation associated with vesicles in the mouth, ruptured vesicles (fluid-filled blisters);


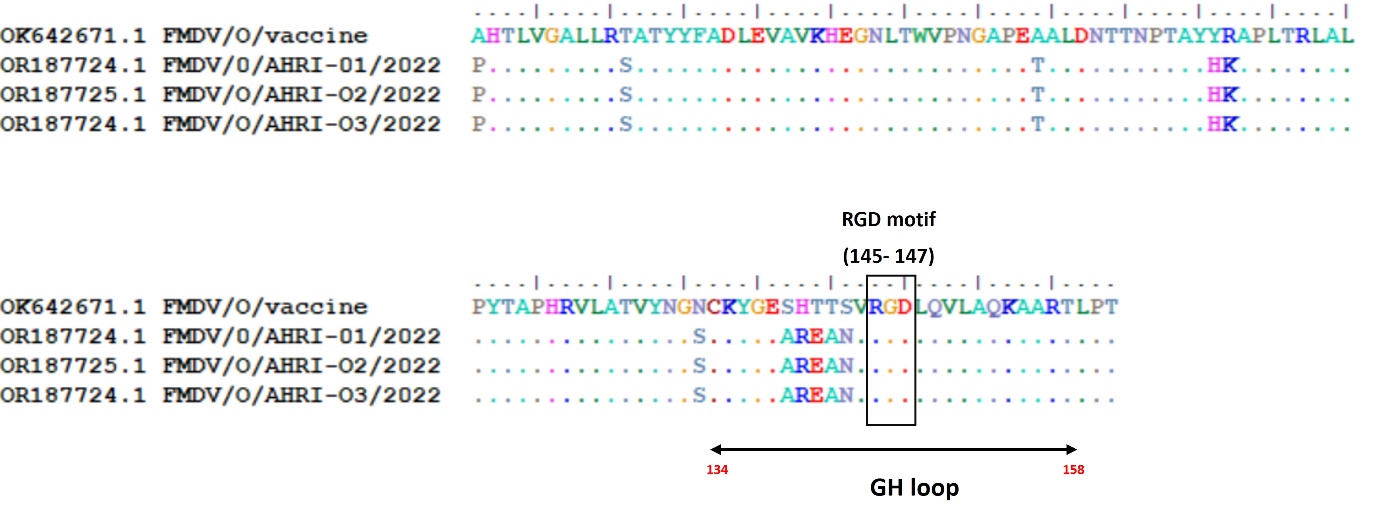


Fig. S2. Deduced amino acid sequence alignment of VP1 of the FMDV serotype O isolates in the present study compared with the FMDV serotype O local vaccine.


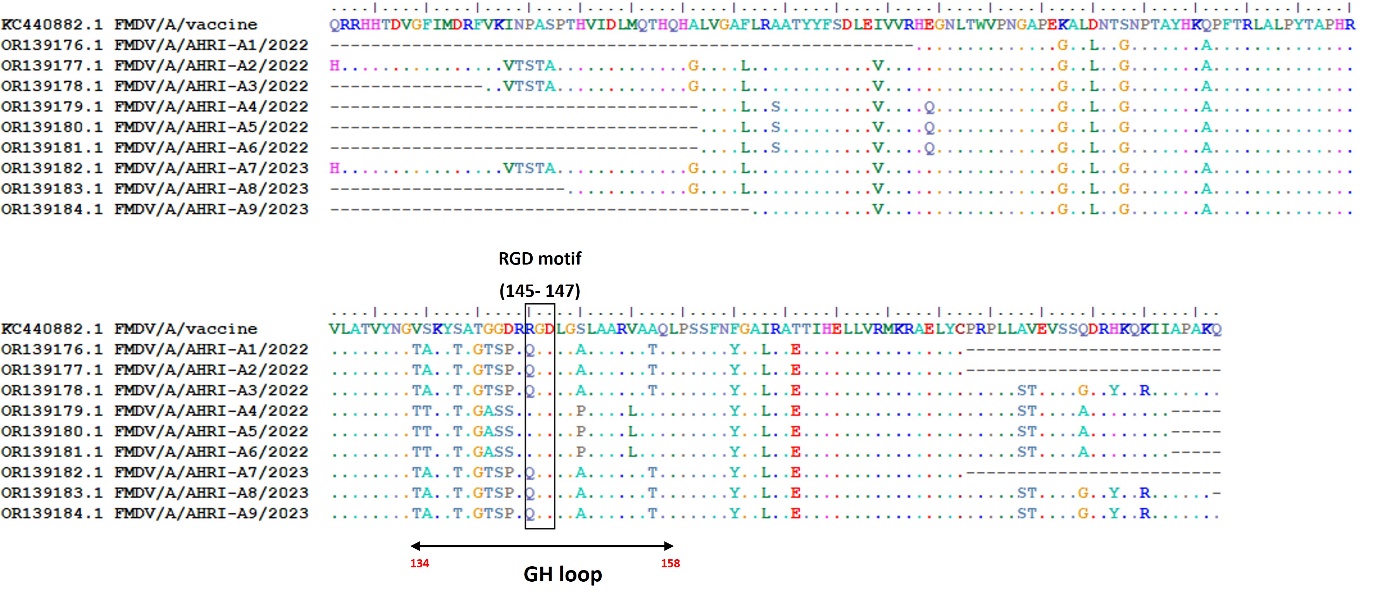


Fig. S3. Deduced amino acid sequence alignment of VP1 of the FMDV serotype A isolates in the present study compared with the FMDV serotype A local vaccine.


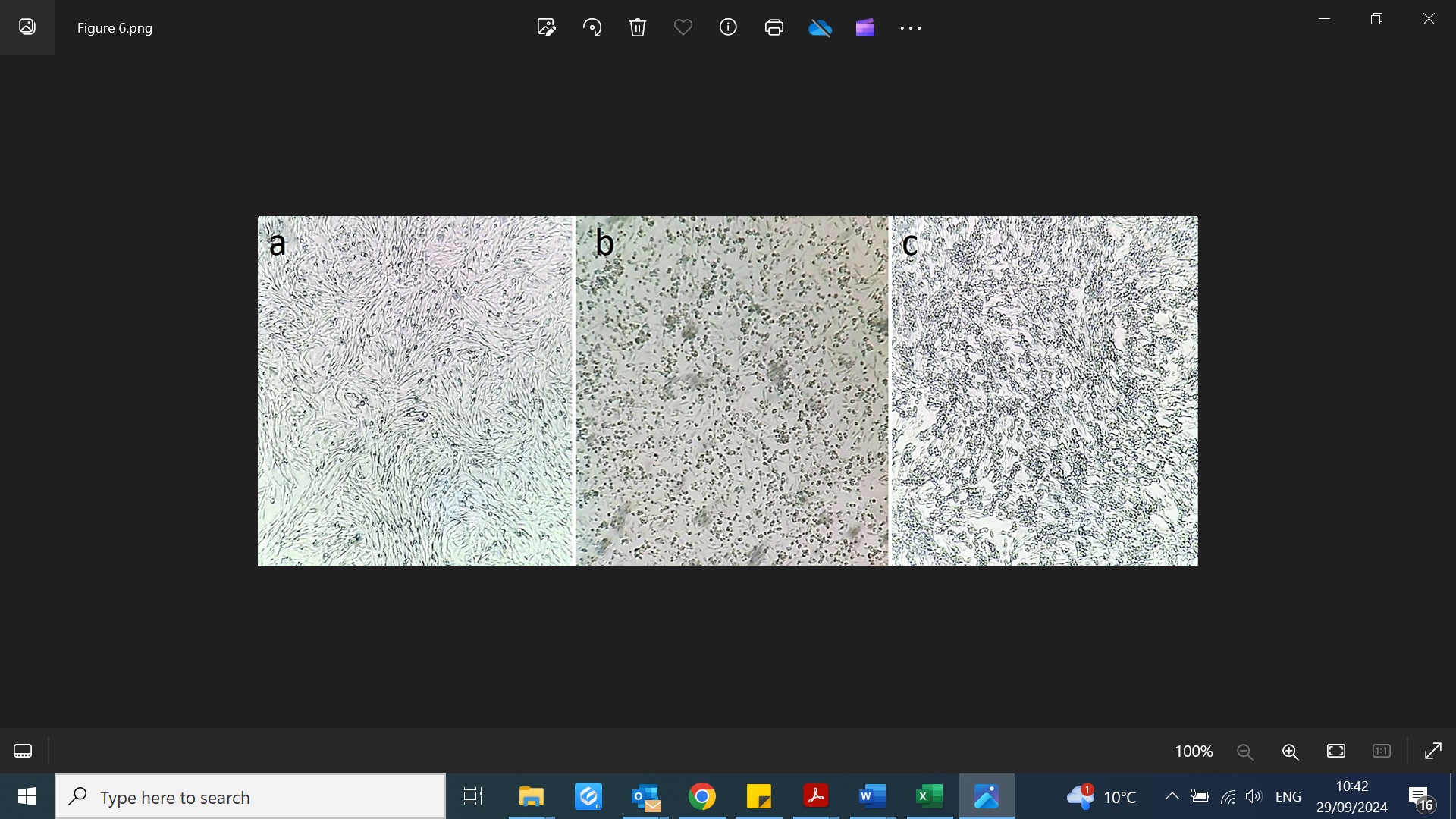


**Fig. S4.** CPE following FMDV strain FMDV/A/AHRI-A7/2023 (OR139182.1) infection on BHK-21 cells after 18 h (a) and after 48 h (b) compared to mock-infected cells (c).

Table S1. Sampling data and prevalence of FMDV in different geographical regions in Egypt during 2022–2023

| **Sample ID** | **Accession number** | **Sample type** | **Species** | **Serotype** | **Location** | **RT-qPCR** |
| --- | --- | --- | --- | --- | --- | --- |
| 1 |  | Epithelium | Cattle | Serotype O | Monofiya | Positive |
| 2 |  | Epithelium | Cattle | Serotype A |  | Positive |
| 3 |  | Epithelium | Cattle | Serotype O |  | Positive |
| 4 |  | Epithelium | Buffalo | Negative |  | Negative |
| 5 |  | Epithelium | Buffalo | Negative |  | Negative |
| 6 |  | Epithelium | Buffalo | Serotype O |  | Positive |
| 7 |  | Epithelium | Buffalo | Serotype O |  | Positive |
| 8 |  | Epithelium | Cattle | Serotype O |  | Positive |
| 9 |  | Epithelium | Cattle | Serotype A |  | Positive |
| 10 |  | Epithelium | Cattle | Serotype A |  | Positive |
| 11 |  | Heart | Cattle | Negative |  | Negative |
| 12 |  | Epithelium | Buffalo | Serotype A |  | Positive |
| 13 |  | Epithelium | Cattle | Serotype A |  | Positive |
| 14 |  | Tongue | Buffalo | Negative |  | Negative |
| 15 |  | Heart | Buffalo | Serotype O |  | Positive |
| 16 |  | Epithelium | Cattle | Negative |  | Negative |
| 17 |  | Epithelium | Cattle | Serotype A |  | Positive |
| 18 |  | Epithelium | Buffalo | Serotype A |  | Positive |
| 19 |  | Epithelium | Buffalo | Serotype A |  | Positive |
| 20 |  | Epithelium | Cattle | Serotype A |  | Positive |
| 21 |  | Heart | Cattle | Negative |  | Negative |
| 22 |  | Epithelium | Cattle | Negative |  | Negative |
| 23 |  | Tongue | Buffalo | Serotype O |  | Positive |
| 24 |  | Heart | Buffalo | Serotype A |  | Positive |
| 25 |  | Epithelium | Buffalo | Serotype A | Beheira | Positive |
| 26 |  | Epithelium | Cattle | Serotype A |  | Positive |
| 27 |  | Epithelium | Buffalo | Serotype A |  | Positive |
| 28 | OR187724.1 | Epithelium | Cattle | Serotype O |  | Positive |
| 29 |  | Epithelium | Cattle | Serotype A |  | Positive |
| 30 | OR139176.1 | Epithelium | Cattle | Serotype A |  | Positive |
| 31 |  | Epithelium | Buffalo | Serotype A |  | Positive |
| 32 |  | Epithelium | Cattle | Serotype O |  | Positive |
| 33 |  | Tongue | Buffalo | Serotype A | Gharbia | Positive |
| 34 |  | Epithelium | Buffalo | Negative |  | Negative |
| 35 |  | Heart | Buffalo | Serotype O |  | Positive |
| 36 |  | Epithelium | Buffalo | Negative |  | Negative |
| 37 |  | Tongue | Buffalo | Serotype O |  | Positive |
| 38 |  | Epithelium | Cattle | Serotype O |  | Positive |
| 39 |  | Epithelium | Cattle | Negative |  | Negative |
| 40 | OR139179.1 | Heart | Buffalo | Serotype A |  | Positive |
| 41 |  | Heart | Buffalo | Serotype A |  | Positive |
| 42 |  | Epithelium | Cattle | Negative | Sharqia | Negative |
| 43 |  | Epithelium | Cattle | Serotype A |  | Positive |
| 44 |  | Epithelium | Cattle | Serotype O |  | Positive |
| 45 |  | Epithelium | Cattle | Serotype O |  | Positive |
| 46 |  | Epithelium | Cattle | Serotype O | New Valley | Positive |
| 47 |  | Epithelium | Cattle | Serotype O |  | Positive |
| 48 |  | Epithelium | Cattle | Serotype A |  | Positive |
| 49 |  | Epithelium | Buffalo | Negative |  | Negative |
| 50 |  | Epithelium | Cattle | Serotype A |  | Positive |
| 51 |  | Epithelium | Cattle | Serotype A |  | Positive |
| 52 | OR187725.1 | Epithelium | Cattle | Serotype O |  | Positive |
| 53 |  | Epithelium | Cattle | Serotype O |  | Positive |
| 54 |  | Epithelium | Cattle | Serotype O |  | Positive |
| 55 |  | Epithelium | Cattle | Serotype A |  | Positive |
| 56 |  | Epithelium | Cattle | Serotype A |  | Positive |
| 57 |  | Epithelium | Cattle | Serotype O |  | Positive |
| 58 | OR139177.1 | Epithelium | Cattle | Serotype A |  | Positive |
| 59 |  | Epithelium | Cattle | Serotype O |  | Positive |
| 60 | OR139181.1 | Epithelium | Cattle | Serotype A |  | Positive |
| 61 |  | Epithelium | Cattle | Serotype O |  | Positive |
| 62 |  | Epithelium | Cattle | Serotype A |  | Positive |
| 63 |  | Tongue | Cattle | Serotype A | Sohag | Positive |
| 64 |  | Epithelium | Buffalo | Serotype A |  | Positive |
| 65 |  | Epithelium | Cattle | Serotype A |  | Positive |
| 66 |  | Epithelium | Buffalo | Serotype A |  | Positive |
| 67 |  | Epithelium | Cattle | Serotype A |  | Positive |
| 68 |  | Epithelium | Cattle | Serotype A |  | Positive |
| 69 |  | Epithelium | Cattle | Negative | Minya | Negative |
| 70 |  | Epithelium | Cattle | Serotype A |  | Positive |
| 71 |  | Epithelium | Cattle | Negative |  | Negative |
| 72 |  | Epithelium | Buffalo | Negative |  | Negative |
| 73 |  | Epithelium | Cattle | Serotype A |  | Positive |
| 74 |  | Epithelium | Cattle | Serotype A |  | Positive |
| 75 |  | Epithelium | Cattle | Negative |  | Negative |
| 76 |  | Epithelium | Buffalo | Negative |  | Negative |
| 77 |  | Epithelium | Cattle | Serotype A |  | Positive |
| 78 | OR139178.1 | Epithelium | Buffalo | Serotype A |  | Positive |
| 79 |  | Epithelium | Buffalo | Negative |  | Negative |
| 80 | OR187726.1 | Epithelium | Cattle | Serotype O |  | Positive |
| 81 |  | Epithelium | Cattle | Serotype A |  | Positive |
| 82 |  | Tongue | Buffalo | Negative |  | Negative |
| 83 |  | Epithelium | Cattle | Serotype A |  | Positive |
| 84 | OR139180.1 | Epithelium | Cattle | Serotype A |  | Positive |
| 85 |  | Epithelium | Buffalo | Serotype A |  | Positive |
| 86 |  | Epithelium | Cattle | Serotype O | Luxor | Positive |
| 87 |  | Epithelium | Cattle | Serotype O |  | Positive |
| 88 |  | Epithelium | Buffalo | Serotype A |  | Positive |
| 89 |  | Epithelium | Cattle | Serotype A | Aswan | Positive |
| 90 |  | Epithelium | Buffalo | Serotype A |  | Positive |
| 91 |  | Heart | Cattle | Serotype O |  | Positive |
| 92 |  | Epithelium | Buffalo | Serotype O |  | Positive |
| 93 |  | Tongue | Cattle | Serotype A | Beni-Suef | Positive |
| 94 |  | Tongue | Cattle | Serotype A |  | Positive |
| 95 |  | Epithelium | Cattle | Serotype A |  | Positive |
| 96 |  | Epithelium | Cattle | Serotype A |  | Positive |
| 97 |  | Epithelium | Cattle | Negative | Monofiya | Negative |
| 98 |  | Heart | Buffalo | Serotype A |  | Positive |
| 99 |  | Tongue | Buffalo | Serotype A |  | Positive |
| 100 |  | Heart | Buffalo | Serotype A |  | Positive |
| 101 |  | Tongue | Cattle | Serotype A |  | Positive |
| 102 |  | Heart | Cattle | Serotype A |  | Positive |
| 103 |  | Tongue | Cattle | Negative |  | Negative |
| 104 |  | Heart | Cattle | Negative |  | Negative |
| 105 |  | Tongue | Cattle | Serotype A |  | Positive |
| 106 | OR139182.1 | Epithelium | Buffalo | Serotype A |  | Positive |
| 107 |  | Heart | Buffalo | Negative |  | Negative |
| 108 |  | Epithelium | Cattle | Negative | Beheira | Negative |
| 109 |  | Heart | Cattle | Negative |  | Negative |
| 110 |  | Epithelium | Cattle | Negative |  | Negative |
| 11 |  | Epithelium | Cattle | Negative |  | Negative |
| 112 |  | Epithelium | Cattle | Negative | Gharbia | Negative |
| 113 |  | Epithelium | Buffalo | Negative |  | Negative |
| 114 |  | Epithelium | Cattle | Negative |  | Negative |
| 115 |  | Tongue | Buffalo | Negative |  | Negative |
| 116 |  | Epithelium | Cattle | Negative |  | Negative |
| 117 |  | Epithelium | Cattle | Serotype A | Sharqia | Positive |
| 118 |  | Epithelium | Buffalo | Negative |  | Negative |
| 119 |  | Epithelium | Buffalo | Negative |  | Negative |
| 120 |  | Epithelium | Cattle | Negative | New Valley | Negative |
| 121 |  | Epithelium | Cattle | Serotype A |  | Positive |
| 122 | OR139184.1 | Epithelium | Cattle | Serotype A |  | Positive |
| 123 |  | Epithelium | Buffalo | Serotype A | Sohag | Positive |
| 124 |  | Epithelium | Cattle | Negative |  | Negative |
| 125 |  | Epithelium | Cattle | Serotype A |  | Positive |
| 126 |  | Epithelium | Cattle | Negative | Minya | Negative |
| 127 |  | Epithelium | Cattle | Negative |  | Negative |
| 128 |  | Epithelium | Cattle | Serotype A |  | Positive |
| 129 |  | Epithelium | Buffalo | Negative |  | Negative |
| 130 |  | Epithelium | Cattle | Negative |  | Negative |
| 131 |  | Epithelium | Cattle | Negative |  | Negative |
| 132 | OR139183.1 | Tongue | Cattle | Serotype A | Beni-Suef | Positive |
| 133 |  | Epithelium | Buffalo | Negative |  | Negative |
| 134 |  | Epithelium | Cattle | Negative |  | Negative |
